# Genes Associated with Thoracic Aortic Aneurysm and Dissection: 2019 Update and Clinical Implications

**DOI:** 10.1055/s-0039-3400233

**Published:** 2019-12-16

**Authors:** Thais Faggion Vinholo, Adam J. Brownstein, Bulat A. Ziganshin, Mohammad A. Zafar, Helena Kuivaniemi, Simon C. Body, Allen E. Bale, John A. Elefteriades

**Affiliations:** 1 Aortic Institute at Yale-New Haven Hospital, Yale University School of Medicine, New Haven, Connecticut; 2 Department of Medicine, Johns Hopkins Hospital and Johns Hopkins School of Medicine, Baltimore, Maryland; 3 Department of Cardiovascular and Endovascular Surgery, Kazan State Medical University, Kazan, Russia; 4 Division of Molecular Biology and Human Genetics, Department of Biomedical Sciences, and Department of Psychiatry, Faculty of Medicine and Health Sciences, Stellenbosch University, Tygerberg, South Africa; 5 Department of Anesthesia, Critical Care and Pain Medicine, Beth Israel Deaconess Medical Center, Harvard Medical School, Boston, Massachusetts; 6 Department of Genetics, Yale School of Medicine, New Haven, Connecticut

**Keywords:** genetics, thoracic aortic aneurysm, aortic dissection

## Abstract

Thoracic aortic aneurysm is a typically silent disease characterized by a lethal natural history. Since the discovery of the familial nature of thoracic aortic aneurysm and dissection (TAAD) almost 2 decades ago, our understanding of the genetics of this disorder has undergone a transformative amplification. To date, at least 37 TAAD-causing genes have been identified and an estimated 30% of the patients with familial nonsyndromic TAAD harbor a pathogenic mutation in one of these genes. In this review, we present our yearly update summarizing the genes associated with TAAD and the ensuing clinical implications for surgical intervention. Molecular genetics will continue to bolster this burgeoning catalog of culprit genes, enabling the provision of personalized aortic care.

## Introduction

**Fig. 1:**
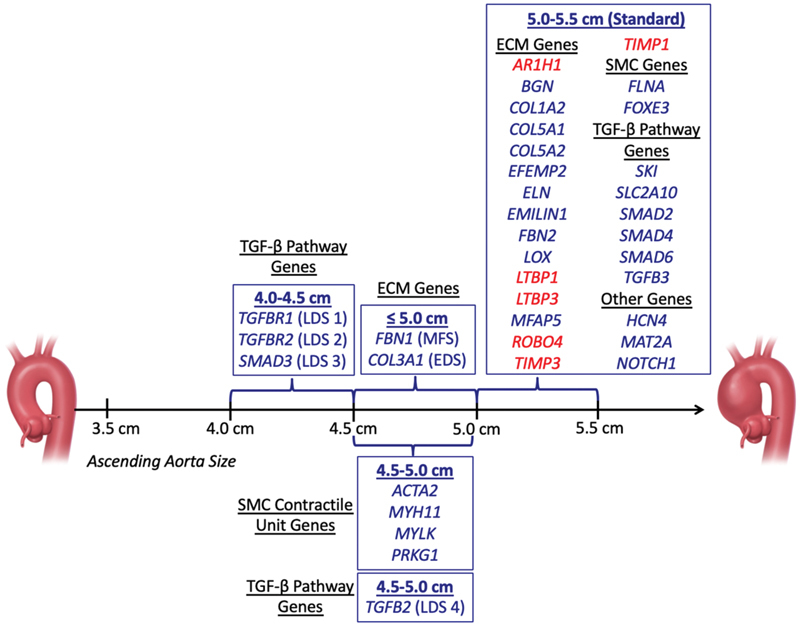
Ascending aortic dimensions for prophylactic surgical intervention (Data derived from Table 1 and modified with permission from Brownstein et al 2 ). Any gene newly reported during the past year to be associated with TAAD is highlighted in red. ECM, extracellular matrix; SMC, smooth muscle cell; TGF, transforming growth factor.

This review presents an annual update to the article “Genes Associated with Thoracic Aortic Aneurysm and Dissection: Update and Clinical Implications” originally published in 2017 and updated in 2018 in AORTA. 1 2 We have updated the list of genes with identified genetic variants predisposing individuals to a thoracic aortic aneurysm or dissection (TAAD) in Table 1 , and the recommendation for individualized surgical interventions for specific genetic mutations is presented in Fig. 1 .

**Table 1:** Genes associated with syndromic and nonsyndromic thoracic aortic aneurysm and/or dissection, associated vascular characteristics, and size criteria for elective surgical intervention (any gene newly reported during the past year to be associated with TAAD is highlighted in red)

Gene	Protein	Animal model leading to vascular phenotype?	Syndromic TAAD	Nonsyndromic FTAAD	Associated disease/syndrome	Associated clinical characteristics of the vasculature	Ascending aorta size (cm) for surgical intervention	Mode of inheritance	OMIM
*ACTA2*	Smooth muscle α-actin	Yes 20	+	+	AAT6 + multisystemic smooth muscle dysfunction + MYMY5	TAAD, early aortic dissection ^t^ , CAD, stroke (moyamoya disease), PDA, pulmonary artery dilation, BAV 21 22	4.5–5.0 a 23 24 25	AD	611788 613834 614042
*ARIH1*	Ariadne RBR E3 ubiquitin protein ligase 1 15	No	+	+	FTAA	Aortic and intracranial aneurysm 15	Standard	Unknown	605624
*BGN*	Biglycan	Yes 26	+	−	Meester-Loeys syndrome	ARD, TAAD, pulmonary artery aneurysm, IA, arterial tortuosity 27	Standard	X-linked	300989
*COL1A2*	Collagen 1 α2 chain	No	+	−	EDS, arthrochalasia Type (VIIb) + cardiac valvular type	Borderline aortic root enlargement 22 28	Standard	AD + AR	130060 225320
*COL3A1*	Collagen 3 α1 chain	Yes 29 30	+	−	EDS, vascular Type (IV)	TAAD, early aortic dissection ^t^ , visceral arterial dissection, vessel fragility, IA 31 32 33	5.0 b 33	AD	130050
*COL5A1*	Collagen 5 α1 chain	No e	+	−	EDS, classic Type I	ARD, rupture/dissection of medium-sized arteries 34 35 36	Standard	AD	130000
*COL5A2*	Collagen 5 α2 chain	Partially f	+	−	EDS, classic Type II	ARD 37	Standard	AD	130000
*EFEMP2*	Fibulin-4	Yes 38 39	+	−	Cutis laxa, AR Type Ib	Ascending aortic aneurysms, other arterial aneurysms, arterial tortuosity, and stenosis 40	Standard	AR	614437
*ELN*	Elastin	No	+	−	Cutis laxa, AD	ARD, ascending aortic aneurysm and dissection, BAV, IA possibly associated with SVAS 41 42 43	Standard	AD	123700 185500
*EMILIN1*	Elastin microfibril interfacer 1	No	+	−	CTD and peripheral neuropathy	Ascending and descending aortic aneurysm 44	Standard	AD	Unassigned
*FBN1*	Fibrillin-1	Yes 45 46 47 48 49	+	+	Marfan syndrome	ARD, TAAD, AAA, other arterial aneurysms, pulmonary artery dilatation, arterial tortuosity 50	5.0 25 51	AD	154700
*FBN2*	Fibrillin-2	No	+	−	Contractural arachnodactyly	Rare ARD and aortic dissection, 52 BAV, PDA	Standard	AD	121050
*FLNA*	Filamin A	Yes 53 54	+	−	Periventricular nodular heterotopia and otopalatodigital syndrome	Aortic dilatation/aneurysms, peripheral arterial dilatation, 55 PDA, IA, 56 BAV	Standard	XLD	300049
*FOXE3*	Forkhead box 3	Yes 57	−	+	AAT11	TAAD (primarily Type A dissection) 57	Standard	AD	617349
*HCN4*	Hyperpolarization-activated cyclic nucleotide-gated potassium channel 4	No	−	+	Noncompaction cardiomyopathy, bradycardia, and mitral valve disease	Ascending aorta dilation 58	Standard	AD	163800
*LOX*	Lysyl oxidase	Yes 59 60 61 62	−	+	AAT10	TAAD, AAA, hepatic artery aneurysm, BAV, CAD	Standard	AD	617168
* LTBP1 g*	Latent TGF-β binding protein	No h 10	+	−	Aortic dilation with associated musculoskeletal findings	TAAD 10	Standard	AD	150390
*LTBP3*	Latent TGF-β binding protein	Yes i 12	+	−	Dental anomalies and short stature	TAAD, AAA, visceral and peripheral arterial aneurysm 12	Standard	AR	602090
*MAT2A*	Methionine adenosyltransferase II α	No j 63	−	+	FTAA	Thoracic aortic aneurysms, BAV 63	Standard	AD	Unassigned
*MFAP5*	Microfibril-associated glycoprotein 2	Partially k 64	−	+	AAT9	ARD, TAAD 65	Standard	AD	616166
*MYH11*	Smooth muscle myosin heavy chain	Partially l 66	−	+	AAT4	TAAD, early aortic dissection ^t^ , PDA, CAD, peripheral vascular occlusive disease, carotid IA 67	4.5–5.0 25 67	AD	132900
*MYLK*	Myosin light chain kinase	No m 68	−	+	AAT7	TAAD, early aortic dissections ^t^ , 17 69 70	4.5–5.0 a 25 68	AD	613780
*NOTCH1*	NOTCH1	Partially n	−	+	AOVD1	BAV/TAAD 71 72	Standard	AD	109730
*PRKG1*	Type I cGMP-dependent protein kinase	No	−	+	AAT8	TAAD, early aortic dissection ^t^ , AAA, coronary artery aneurysm/dissection, aortic tortuosity, small vessel CVD	4.5–5.0 73	AD	615436
*ROBO4*	Roundabout guidance receptor 4	Yes	−	+	BAV	BAV/TAA 9	Standard	AD	607528
*SKI*	Sloan Kettering proto-oncoprotein	No o	+	−	Shprintzen-Goldberg syndrome	ARD, arterial tortuosity, pulmonary artery dilation, other (splenic) arterial aneurysms 74	Standard	AD	182212
*SLC2A10*	Glucose transporter 10	No p	+	−	Arterial tortuosity syndrome	ARD, 75 ascending aortic aneurysms, 75 other arterial aneurysms, arterial tortuosity, elongated arteries aortic/pulmonary artery stenosis	Standard	AR	208050
*SMAD2*	SMAD2	No	+	−	Unidentified CTD with arterial aneurysm/dissections	ARD, ascending aortic aneurysms, vertebral/carotid aneurysms and dissections, AAA 76 77	Standard	AD	Unassigned
*SMAD3*	SMAD3	Partially q 78	+	+	LDS Type III	ARD, TAAD, early aortic dissection ^t^ , AAA, arterial tortuosity, other arterial aneurysms/dissections, IA, BAV 79 80	4.0–4.2 25 51	AD	613795
*SMAD4*	SMAD4	Yes 81	+	−	JP/HHT syndrome	ARD, TAAD, AVMs, IA 82 83	Standard	AD	175050
*SMAD6*	SMAD6	No r	−	+	AOVD2	BAV/TAA 84	Standard	AD	602931
*TIMP3*	Tissue inhibitors of matrix metalloproteinase 3	No	+	−	AOVD	BAV/TAA 16	Standard	XLD	188826
*TIMP1*	Tissue inhibitors of matrix metalloproteinase 1	No	−	−	AOVD	BAV/TAA 16	Standard	XLD	305370
*TGFB2*	TGF-β2	Yes 85	+	+	LDS Type IV	ARD, TAAD, arterial tortuosity, other arterial aneurysms, BAV 85 86	4.5–5.0 c 87	AD	614816
*TGFB3*	TGF-β3	No s	+	−	LDS Type V	ARD, TAAD, AAA/dissection, other arterial aneurysms, IA/dissection 88	Standard	AD	615582
*TGFBR1*	TGF-β receptor type I	Yes 89	+	+	LDS Type I + AAT5	TAAD, early aortic dissection ^t^ , AAA, arterial tortuosity, other arterial aneurysms/dissection, IA, PDA, BAV 90	4.0–4.5 d 25 51 91	AD	609192
*TGFBR2*	TGF-β receptor Type II	Yes 81 89	+	+	LDS Type II + AAT3	TAAD, early aortic dissection ^t^ , AAA, arterial tortuosity, other arterial aneurysms/dissection, IA, PDA, BAV 90	4.0–4.5 d 25 51 91	AD	610168

Abbreviations: AAA, abdominal aortic aneurysm; AAT, aortic aneurysm, familial thoracic; AD, autosomal dominant; AOVD, aortic valve disease; AR, autosomal recessive; ARD, aortic root dilatation; AVM, arteriovenous malformation; BAV, bicuspid aortic valve; CAD, coronary artery disease; CTD, connective tissue disease; CVD, cerebrovascular disease; EDS, Ehlers-Danlos syndrome; FTAA, familial thoracic aortic aneurysm; FTAAD, familial thoracic aortic aneurysm and/or dissection; HHT, hereditary hemorrhagic telangiectasia; IA, intracranial aneurysm; JP, juvenile polyposis; LDS, Loeys-Dietz syndrome; MYMY, moyamoya disease; OMIM, Online Mendelian Inheritance in Man; PDA, patent ductus arteriosus; SVAS, supravalvular aortic stenosis; TGF, transforming growth factor; TAAD, thoracic aortic aneurysm and/or dissection; TGFBR, TGF-β receptor; XLD, X-linked dominant.

Note: It is important to note that since mutations in many of these genes are rare and have only recently been implicated in TAAD, there is a lack of adequate prospective clinical studies. Therefore, it is difficult to establish threshold diameters for the intervention of TAAs, and each individual must be considered on a case by case basis, taking into account the rate of change in aneurysm size (>0.5 cm per year is considered rapid), any family history of aortic dissection at diameters< 5.0 cm, and the presence of significant aortic regurgitation, which are all indications for early repair if present; A “ + ” symbol in the syndromic TAAD column indicates that mutations in the gene have been found in patients with syndromic TAAD (same for the nonsyndromic TAAD column). A “ − ” symbol in the syndromic TAAD column indicates that mutations in the gene have not been found in patients with syndromic TAAD (same for the nonsyndromic TAAD column); A reference is provided for each of the associated vascular characteristics not reported in the OMIM entry for that gene.

Standard = surgical intervention at 5.0–5.5 cm; Early aortic dissection ^t^ = dissection at aortic diameters <5.0 cm.

a Individuals with MYLK and ACTA2 mutations have been shown to have aortic dissections at a diameter of 4.0 cm. 23 68

b There are no data to set threshold diameters for surgical intervention for EDS Type IV. 51 The Canadian guidelines recommend surgery for aortic root sizes of 4.0–5.0 cm and ascending aorta sizes of 4.2–5.0 cm, though these patients are at high risk of surgical complications due to poor quality vascular tissue. 92

c There are limited data concerning the timing of surgical intervention for LDS Type IV. However, there has been a case of a Type A aortic dissection at an aortic diameter <5.0 cm, 87 hence the recommended threshold range of 4.5–5.0 cm.

d Current U.S. guidelines recommend prophylactic surgery for LDS Types I and II at ascending aortic diameters of 4.0–4.2 cm. 25 51 However, the European guidelines state that more clinical data are required. 33 Patients with TGFBR2 mutations have similar outcomes to patients with FBN1 mutations once their disease is diagnosed, 93 and the clinical course of LDS 1 and 2 does not appear to be as severe as originally reported. 91 94 95 Therefore, medically treated adult patients with LDS 1 or 2 may not require prophylactic surgery at ascending aortic diameters of 4.0–4.2 cm. 21 Individuals with TGFBR2 mutations are more likely to have aortic dissections at diameters <5.0 cm than those with TGFBR1 mutations. 91 95 A more nuanced approach proposed by Jondeau et al utilizing the presence of TGFBR2 mutations (vs. TGFBR1 mutations), the co-occurrence of severe systemic features (arterial tortuosity, hypertelorism, wide scarring), female gender, low body surface area, and a family history of dissection or rapid aortic root enlargement, which are all risk factors for aortic dissection, may be beneficial for LDS 1 and 2 patients to avoid unnecessary surgery at small aortic diameters. 91 Therefore, in LDS 1 or 2 individuals without the above features, Jondeau et al maintain that 4.5 cm may be an appropriate threshold, but females with TGFBR2 mutations and severe systemic features may benefit from surgery at 4.0 cm. 91

e Wenstrup et al found that mice heterozygous for an inactivating mutation in Col5a1 exhibit decreased aortic compliance and tensile strength relative to wild type mice. 96

f Park et al recently demonstrated that Col5a2 haploinsufficiency increased the incidence and severity of AAA and led to aortic arch ruptures and dissections in an angiotensin II-induced aneurysm mouse model. 37 In an earlier paper, Park et al illustrated that mice heterozygous for a null allele in Col5a2 exhibited increased aortic compliance and reduced tensile strength compared with wild type mice. 97

g Chromosome 2p22 deletion.

h Todorovic et al 98 showed that LTBP1 plays an important role in cardiac and bone development. Knockout mice displayed interrupted aortic arch, patent truncus arteriosus, hyperplastic semilunar valves, and atrial sept defects. However, aortic measurements were not mentioned. 10

i Guo et al showed that the knockout mice have larger aortic roots and ascending aortas than wild type, however, no aneurysms or dissections were reported.

j Guo et al found that the knockdown of MAT2AA in zebrafish led to defective aortic arch development. 63

k Combs et al demonstrated that MFAP2 and MFAP5 double knockout (MFAP2 ^−/−^ ;MFAP5 ^−/−^ ) mice exhibit age-dependent aortic dilation, though this is not the case with MFAP5 single knockout mice.

l While Kuang et al reported that a mouse knock-in model (Myh11 ^R247C/R247C^ ) does not lead to a severe vascular phenotype under normal conditions, 99 Bellini et al demonstrated that induced hypertension in this mouse model led to intramural delaminations (separation of aortic wall layers without dissection) or premature deaths (due to aortic dissection based on necroscopy according to unpublished data by Bellini et al) in over 20% of the R247C mice, accompanied by focal accumulation of glycosaminoglycans within the aortic wall (a typical histological feature of TAAD).

m Wang et al demonstrated that SMC-specific knockdown of Mylk in mice led to histopathological changes (increased pools of proteoglycans) and altered gene expression consistent with medial degeneration of the aorta, though no aneurysm formation was observed.

n Koenig et al recently found that Notch1 haploinsufficiency exacerbates the aneurysmal aortic root dilation in a mouse model of MFS and that Notch1 heterozygous mice exhibited aortic root dilation, abnormal smooth muscle cell morphology, and reduced elastic laminae. 100

o Doyle et al found that knockdown of paralogs of mammalian SKI in zebrafish led to craniofacial and cardiac anomalies, including failure of cardiac looping and malformations of the outflow tract. 74 Berk et al showed that mice lacking Ski exhibit craniofacial, skeletal muscle, and central nervous system abnormalities, which are all features of Shprintzen-Goldberg syndrome, but no evidence of aneurysm development was reported. 101

p Mice with homozygous missense mutations in Slc2a10 have not been shown to have the vascular abnormalities seen with arterial tortuosity syndrome, 102 though Cheng et al did demonstrate that such mice do exhibit abnormal elastogenesis within the aortic wall. 103

q Tan et al demonstrated that SMAD3 knockout mice only developed aortic aneurysms with angiotensin II-induced vascular inflammation, though the knockout mice did have medial dissections evident on histological analysis of their aortas and exhibited aortic dilatation relative to wild type mice prior to angiotensin II infusion. 78

r *Galvin et al demonstrated that Madh6, which encodes* SMAD6, *mutant mice exhibited defects in cardiac valve formation, outflow tract septation, vascular tone, and ossification but no aneurysm development was observed.* 104

s *TGFB3 knockout mice die at birth from cleft palate,* 88 *but minor differences in the position and curvature of the aortic arches of these mice compared with wild type mice have been described.* 105

Thoracic aortic aneurysm (TAA) affects 1% of the general population 3 and its natural history is to enlarge an average of 0.14 cm per year. 4 Prior to often lethal dissection or rupture, TAAs are usually asymptomatic. However, if identified and treated with appropriate blood pressure control and surgical intervention, life expectancy is improved.

Report of inherited TAAD in the 1990s 5 has led to the discovery and understanding of genetic and molecular mechanisms of TAAD. 6 To date, variants in 37 genes have been associated with TAAD ( Table 1 ; Fig. 1 ). These genes explain approximately 30% of the familial nonsyndromic TAAD. 7 These genes encode proteins of the extracellular matrix, vascular smooth muscle cell contractile unit, or transforming growth factor β (TGF-β)-signaling pathways 8 and thus are essential to the structure and maintenance of the aortic wall.

During 2018, several important studies were published that have enhanced our understanding of the pathogenesis of TAAD. Gould et al 9 performed whole-exome sequencing (WES) and targeted sequencing on 736 individuals with bicuspid aortic valve (BAV), non-syndromic ascending aortic aneurysm (AscAA), and 376 controls. 9 In 13 (1.8%) of the affected individuals a heterozygous *ROBO4* mutation was identified, including two variants that segregated with disease among two affected families. 9 *ROBO4* is well expressed in vascular endothelial cells and plays a role in endothelial barrier function. 9 In this study, its expression was found to be diminished in the resected aorta sample of an affected individual with AscAA. 9 To further test their hypothesis that *ROBO4* variants lead to the disruption of endothelial performance at a cellular level, thus altering vascular permeability, the authors cultured human aortic endothelial cells and either silenced *ROBO4* or expressed *ROBO4* variants. They confirmed that *ROBO4* abnormalities did indeed induce endothelial barrier dysfunction. Lastly, the authors created homozygous *ROBO4* knockout mice and a knock-in mouse with an *ROBO4* splice donor site mutation; the affected mice presented with a mix of aortic valve dysfunction (BAV and/or aortic regurgitation or stenosis) and AscAA, confirming their suspicion that a heterozygous mutation in *ROBO4* can lead to a nonsyndromic presentation of BAV/AscAA. 9

Latent transforming growth factor binding proteins (LTBP), a family of extracellular matrix glycoproteins, have been shown to play a significant role in TGF-β regulation. 10 LTBP1, in particular, can bind to fibrillin-1 and inactivate TGF-β. 10 11 Quiñones-Pérez et al described a case series involving a three-generation family with TAA found to have a chromosome 2p22.3-p22.2 deletion involving *LTBP1* , amongst other genes. 10 Despite multiple genes being involved in the deletion, *LTBP1* was considered the likely culprit given its relationship to TGF-β. In addition to TAA, the affected individuals displayed additional features of Marfan syndrome (MFS) and Loeys-Dietz syndrome, even though none of them met the criteria for diagnosis.

Mutations of the latent TGF-β binding protein-3 ( *LTBP3* ) gene have been associated with TAAD in a WES study of 271 individuals from unrelated families with heritable thoracic aortic disease (multiple affected family members) without a known genetic etiology for aortopathy. 12 In this study, compound heterozygous variants in one family and a homozygous insertion/deletion variant in *LTBP3* in a second family were identified. Sequencing of 338 additional individuals with non-syndromic TAAD found nine additional heterozygous *LTBP3* rare variants. The authors also demonstrated that *LTBP3* knockout mice manifested enlarged aortic roots and ascending aortas compared with wild type mice. These findings demonstrate that individuals with *LTPB3* are at increased risk for TAAD, in addition to the already established risk for skeletal and dental abnormalities. 12 13 14

Rare mutations in the Parkin-like E3 ubiquitin ligase Ariadne-1 ( *ARIH1* ) have been observed in patients with early-onset or familial TAAD. 15 *AR1H1* encodes a protein of the LINC (linker of nucleoskeleton and cytoskeleton), a protein complex essential for anchoring myocyte nuclei to the cytoskeleton. 15 Aortic tissues from patients with these mutations exhibit affected nuclear morphology in vascular smooth muscle cells.

It is well known there is an increased risk for BAV and TAA among individuals with Turner syndrome, although the precise etiology has thus far remained elusive. Corbitt et al 16 demonstrated that Turner syndrome patients with putatively-deleterious mutations in *TIMP3* are associated with a greater incidence of BAV and TAA than the patients without *TIMP3* variants. Hemizygosity for coincident *TIMP1/TIMP3* variants, synergistically increased the risk for BAV and TAA, 16 due to TIMP1's functional redundancy with *TIMP3.*

Numerous mutations of the myosin light chain kinase ( *MYLK* ) gene have been associated with TAAD. Shalata et al have identified an additional *MYLK* missense mutation in a single pedigree. 17 Myosin light chain kinase phosphorylates myosin regulatory light chains to facilitate actin-myosin generation of contraction. The mutation was shown to be functional, reducing kinase activity.

Insights to the pathogenesis of TAAD are as important as identifying TAAD variants. Nogi et al 18 found the protein expression of small GTP-binding protein GDP dissociation stimulator (SmgGDS) in aortic smooth muscle cells was decreased in TAAD patients compared with controls. 18 SmgGDS is encoded by the *RAP1GDS1* gene and known to be involved in the contraction of vascular smooth muscle cell (VSMC). 18 Using a heterozygous SmgGDS ^+/−^ mouse model, since the complete knockout (SmgGDS ^−/−^ ) was embryologically lethal, they observed that the downregulation of SmgGDS was causing “pathological phenotype changes in VSMC” via the angiotensin-II pathway. 18 Furthermore, they demonstrated that when SmgGDS was overexpressed in the SmgGDS ^+/−^ , the mice had less aortic growth and fewer aortic ruptures, suggesting that SmgGDS could be used as a biomarker or a therapeutic agent.

## Conclusion

Advances in 2018 have increased our understanding of the pathogenesis of TAAD. The number of genes with genetic variants or mutations associated with TAAD has increased from 29 in our original 2017 report 2 to 37 in this 2019 update. Advances in genetic techniques and bioinformatics tools have enabled rapid progress in the genetic and molecular understanding of TAA. As the cost for genome sequencing decreases, we anticipate accelerating progress. With our greater understanding of the genetics of the individuals affected with TAAD and their specific genetic mutations or susceptibility variants, we can provide a personalized aortic care, tailoring surgical recommendations for each patient depending on their individual genetic profiles. Because most families that have multiple affected members with TAAD still have not had known genetic variants identified in the aortopathy genes, we expect many new genes harboring variants for TAAD will be discovered in the foreseeable future and thereby enhance our genetic dictionary. Furthermore, it is important to remind ourselves that every disease-causing mutation starts out as a variant of unknown significance (VUS). 19 Only after extensive functional studies it is possible to confidently state that a VUS is a disease-causing mutation. Such work requires multidisciplinary collaboration.

We will continue to report annual updates regarding the “TAA genetic dictionary” with updates to the Table and Figure below and provide suggested surgical intervention criteria for each identified mutation.
